# Development of fungal-mediated soil suppressiveness against *Fusarium* wilt disease via plant residue manipulation

**DOI:** 10.1186/s40168-021-01133-7

**Published:** 2021-10-12

**Authors:** Xianfu Yuan, Shan Hong, Wu Xiong, Waseem Raza, Zongzhuan Shen, Beibei Wang, Rong Li, Yunze Ruan, Qirong Shen, Francisco Dini-Andreote

**Affiliations:** 1 Jiangsu Provincial Key Lab of Solid Organic Waste Utilization, Jiangsu Collaborative Innovation Center of Solid Organic Wastes, Educational Ministry Engineering Center of Resource-saving fertilizers, Nanjing Agricultural University, Nanjing, 210095 Jiangsu People’s Republic of China; 2 The Key Laboratory of Plant Immunity, Nanjing Agricultural University, Nanjing, 210095 Jiangsu People’s Republic of China; 3 Hainan Key Laboratory for Sustainable Utilization of Tropical Bio-resources, College of Tropical Crops, Hainan University, Haikou, 570228 People’s Republic of China; 4 Ecology and Biodiversity Group, Department of Biology, Institute of Environmental Biology, Utrecht University, 3584 CH Utrecht, the Netherlands; 5 Department of Plant Science, The Pennsylvania State University, University Park, PA USA; 6 Huck Institutes of the Life Sciences, The Pennsylvania State University, University Park, PA USA

**Keywords:** *Fusarium* wilt disease, Crop rotation, Plant residues, *Fusarium solani*, *Aspergillus fumigatus*, Disease suppression

## Abstract

**Background:**

The development of suppressive soils is a promising strategy to protect plants against soil-borne diseases in a sustainable and viable manner. The use of crop rotation and the incorporation of plant residues into the soil are known to alleviate the stress imposed by soil pathogens through dynamics changes in soil biological and physicochemical properties. However, relatively little is known about the extent to which specific soil amendments of plant residues trigger the development of plant-protective microbiomes. Here, we investigated how the incorporation of pineapple residues in soils highly infested with the banana *Fusarium* wilt disease alleviates the pathogen pressure via changes in soil microbiomes.

**Results:**

The addition of above- and below-ground pineapple residues in highly infested soils significantly reduced the number of pathogens in the soil, thus resulting in a lower disease incidence. The development of suppressive soils was mostly related to trackable changes in specific fungal taxa affiliated with *Aspergillus fumigatus* and *Fusarium solani*, both of which displayed inhibitory effects against the pathogen. These antagonistic effects were further validated using an in vitro assay in which the pathogen control was related to growth inhibition via directly secreted antimicrobial substances and indirect interspecific competition for nutrients. The disease suppressive potential of these fungal strains was later validated using microbial inoculation in a well-controlled pot experiment.

**Conclusions:**

These results mechanistically demonstrated how the incorporation of specific plant residues into the soil induces trackable changes in the soil microbiome with direct implications for disease suppression. The incorporation of pineapple residues in the soil alleviated the pathogen pressure by increasing the relative abundance of antagonistic fungal taxa causing a negative effect on pathogen growth and disease incidence. Taken together, this study provides a successful example of how specific agricultural management strategies can be used to manipulate the soil microbiome towards the development of suppressive soils against economically important soil-borne diseases.

Video Abstract

**Supplementary Information:**

The online version contains supplementary material available at 10.1186/s40168-021-01133-7.

## Introduction

Identifying the key factors controlling a given function in agroecosystems is often a challenge, and further devising strategies to properly manipulate them to obtain desirable biological benefits can be even more complex [1, 2]. Sustainable intensification in agriculture relies on the development of practices that enhance crop yields without causing adverse environmental impacts [3]. This can be achieved by implementing management that directly and/or indirectly modulates the soil microbiome to reduce adverse biotic pressures caused by pathogens [4, 5]. For instance, it is known that successive cycles of monocultures result in the progressive accumulation of soil-borne pathogens, whereas specific crop rotation systems can alleviate pathogen pressure via changes in soil chemistry and biological properties [6]. However, there is an urgent need to further explore the mechanisms by which agricultural management promotes or enhances soil-borne disease suppression, with a specific focus on inducible and trackable changes in the soil microbiome [7].

Several pathogens causing soil-borne diseases are increasingly threatening agricultural systems worldwide [8], and the development of microbial-mediated soil suppressiveness has been suggested as a promising and sustainable option to alleviate the impact of these diseases [9]. Conceptually, the soil microbiome pathogen density, diversity, and structure are the main factors operating in either promoting disease suppression or incidence [10, 11]. In this scenario, the occurrence of key beneficial microbial taxa in soil has been shown to effectively control soil-borne diseases by directly inhibiting diverse pathogens [12]. These beneficial/protective taxa operate have been reported to operate via (*i*) the production of antimicrobial or toxic substances that directly affect the pathogens, (*ii*) competition for space and resources indirectly suppressing the pathogen growth, and/or (*iii*) the induction of plant-protective responses via systemic metabolic regulation of physiology and immunity [13–15].

Mounting evidence suggests that soil suppressiveness can be prompted by using specific agricultural practices, such as the application of organic materials or biofertilizers, the incorporation of plant residues in the soil, and crop rotation [16, 17]. These strategies mostly rely on promoting the emergence of protective microbiomes and/or directly impacting the population of the pathogen—both of which account for effective disease control [18]. The use of crop rotation systems can favor the dynamic cycling of nutrients in soil and the temporal shifts in the soil microbiome that alleviates the pathogen accumulation in soil [19, 20]. In addition, it has been shown that plant residue decomposition and root exudates in soil can leave a long-lasting effect on pathogen control as a result of crop rotation, a phenomenon termed as ‘legacy effects’ and relieve soil pressure by changing physicochemical properties [21, 22]. As such, it is clear that crop rotation and plant residue manipulation in the field are important strategies for soil-borne disease management. However, these strategies have received relatively little attention, as efforts have mostly been given to investigate how such approaches relate to overall aspects of soil quality, nutrient cycle, and crop performance [23–25]. Hence, understanding how these approaches impact the soil microbiome composition and its ecological function remains elusive [26]. This opens up potential opportunities to explore beneficial outcomes of agricultural management associated with soil-borne disease control [27].

The banana industry is seriously threatened by the *Fusarium* wilt disease, which is caused by the soil-borne fungus *Fusarium oxysporum* f. sp. *cubense* (*Foc*) [28, 29]. During the early period of the last century, the *Fusarium* wilt disease was responsible for wiping out the “Gros Michel” banana industry, caused by the pathogen of *Foc* race 1. This epidemic was effectively controlled by replacing this cultivar by the cultivar “*Cavendish*” [30]. However, a few decades ago, the *Fusarium* wilt disease resurged on the cultivar “*Cavendish*”, at this time caused by the *Fusarium oxysporum* f. sp. *cubense* tropical race 4 (*Foc*TR4) or *Fusarium oxysporum* f. sp. *cubense* subtropical race 4 (*Foc*SR4) [31, 32]. Whereas the *Foc*SR4 has been limited to subtropical climates, *Foc*TR4 has a broader spectrum of occurrence across both subtropical and tropical regions, including the region of Hainan in China [33, 34]. Therefore, the development of effective strategies to control this pathogen in banana field sites is urgently needed. Here, we studied the impact of plant residue incorporation on the development of microbial-mediated soil suppressiveness that effectively controls the incidence of the banana *Fusarium* wilt disease caused by the pathogen *Foc*TR4. For that, we used soils from sites containing a high population of the pathogen and developed a series of experiments and assays to better understand the biological and mechanistic bases of pathogen suppression. We hypothesized the development of disease-suppressive soils to be related to the increase in the abundance of antagonistic fungal taxa that directly compete with *Foc*TR4, and not due to changes in soil chemistry resulting from plant residue incorporation. In particular, we aimed at addressing the following questions: (1) to what extent the incorporation of pineapple residues into the soil reduces the *Foc*TR4 density and the incidence of banana *Fusarium* wilt disease under well-controlled conditions? (2) Are these negative effects on *Foc*TR4 associated with changes in soil biological or physicochemical properties? (3) Are there specific microbial taxa associated with the development of *Foc*TR4 disease suppression? (4) What are the microbial-mediated mechanisms associated with the suppression of *Foc*TR4 in soil?

## Materials and methods

### Soil sampling and crop residues preparation

Soil samples were collected from a field located at the Hainan WanZhong Co., Ltd. in Jianfeng Town, Ledong County, Hainan Province, China (108°45′ E, 18°38′ N). This site has a history of banana monoculture cultivation of 8 years, and it is known to be highly affected by the *Fusarium* wilt disease, which was approximately 60% at the time of soil collection [35]. The soil has a sandy loam texture with a pH value of 6.14, containing 11.37 g kg^−1^ of total nitrogen (TN), 65.06 g kg^−1^ of total carbon (TC), 0.96 g kg^−1^ of total phosphorus (TP), 0.27 g kg^−1^ of total potassium (TK), and 0.12 g kg^−1^ of total magnesium (TMg). Soil samples collected in the field were stored in the shade to perform twice glasshouse experiments (see below).

The banana-pineapple rotation experiment was performed at the same site where soil samples were collected (previously under 8 years of banana monoculture cultivation). We selected this site due to its high incidence of banana *Fusarium* wilt disease. Visually healthy crop residues, i.e., displaying no typical symptoms of crop disease at harvesting (for banana and pineapple), were collected across the monoculture and banana-pineapple rotation treatments. These residues were transferred into plastic packaging bags, kept on ice, and immediately transported to the laboratory (< 6 h). After that, crop residues were carefully washed three times with sterile deionized water in the laboratory, and separated into below- and above-ground plant parts. BS, above-ground banana residue; BR, below-ground banana residue; PS, above-ground pineapple residue; and PR, below-ground pineapple residue. These crop residues were chopped into tiny pieces and ground down to powder. Each residue type was sieved through a 4-mm mesh, and the total nutrient content was measured [36]. See Table S1 for details.

### Experimental design

The glasshouse experiments were performed (experiment 1: October to December 2015; experiment 2: March to June 2016) at the WanZhong Co., Ltd in Ledong County, Hainan Province, China. Together, both experiments consisted of 300 polypropylene pots (18 cm × 25 cm, diameter × height), and each pot was filled with 5 kg of well-mixed pre-sampled soil. Five treatments were established with or without the amendment with the different pre-processed plant residue: above-ground banana residue (BS), below-ground banana residue (BR), above-ground pineapple residue (PS), below-ground pineapple residue (PR), in addition to the control soil without residue (CK). Each pot was supplemented with 100 g of the respective amendment before the banana seedling transplantation (i.e., 2% concentration). This amount is in line with the incorporation of residues in the field settings [37, 38]. Each individual treatment was adjusted to equal amounts of TN, TP, and TK based on the nutrient availability determined for each residue. Adjustments of TN, TP, and TK levels were carried out using mineral fertilizers (see Table S2, for details). The entire experiment was carried out using a complete randomized block design with three replicates per treatment. Each replicate contained 10 individual pots. One banana seedling (Musa AAA *Cavendish* cv. Brazilian) was transplanted in each pot. The seedlings were kindly provided by the Hainan Wan Zhong Co., Ltd.

### Determination of *Fusarium* wilt disease incidence and experimental sampling

Based on typical wilt symptoms associated with banana *Fusarium* disease, the diseased bananas were monitored after 1 month until the number of diseased bananas was stable. Then the evaluation of the *Fusarium* wilt disease was monitored throughout the experiment and quantified as the percentage of infected plants relative to the total number of plants [39]. The symptoms observed were dark brown discoloration for vascular tissues, pseudostem splitting, leaf yellowing and plant death. Likewise, the disease severity was also evaluated based on the typical disease symptoms [39]. In each pot, soil samples were collected at a depth of 5–15 cm with a sterile horticulture shovel after removing the banana plants at the end of the experiment. A total of 50 g of soil in each pot was collected. For each replicate, soil samples from three pots were mixed as a composite sample and each block contained three mixed samples. A total of nine mixed samples were obtained and processed for each treatment. After sieving through a 2-mm mesh, each composite soil sample was divided into two parts. One part was stored at – 80 °C for subsequent DNA extraction, and the other was stored at 4 °C for short-term experiments. All samples collected were subjected to physical chemistry analyses to determine the soil pH, electrical conductivity (EC), available phosphorus (AP), available potassium (AK), total organic carbon (TOC), ammonium (NH_4_^+^) and nitrate (NO_3_^−^), following previously described methods [36].

### Soil DNA extraction and microbiome profiling

Total soil DNA was extracted using the PowerSoil DNA Isolation Kit (MoBio Laboratories Inc., USA), according to the manufacturer’s instructions. The sequencing libraries were constructed as previously described [40, 41]. The primers of ITS1F/ITS2R were used to amplify the ITS1 region of ITS using the Thermo Scientific® Phusion High-Fidelity Polymerase Chain Reaction (PCR) Master Mix (New England Biolabs, UK). Details on the amplification protocol are described by Shen et al. [39]. The amplicon concentration in each sample and the final library quality were measured using an Agilent 2100 Bioanalyser Instrument (Agilent Technologies Co. Ltd., USA) and the KAPA Library Quantification Kit (Kapa Biosystems, USA). Amplicon libraries were sequenced on an Illumina MiSeq 2000 platform at the Personal Biotechnology Company (Shanghai, China).

### Bioinformatic analysis

Sequences were split according to their unique barcodes, and the adaptors and primers were trimmed using the Quantitative Insights into Microbial Ecology (QIIME) [42]. After the removal of low-quality sequences, the forward and reverse sequences of each sample were merged. The sequences retained in each sample were processed using the UPARSE pipeline to generate operational taxonomic units (OTUs) at 99% of nucleotide identity [43]. For the analysis of fungal taxa, representative sequences of each OTU were selected and classified using the UNITE ITS database [44]. All raw sequence data are available at the National Center for Biotechnology Information (NCBI) Sequence Read Archive (SRA) database under the accession number PRJNA670608.

The relative abundances of fungal taxonomic groups in each sample were calculated using the sequence number affiliated to each OTU divided by the total number of sequences. Principal coordinates analysis (PCoA) based on Bray-Curtis distances and the analysis of similarities (ANOSIM) were performed using the *vegan* package in R software [45]. In order to trace significant changes in fungal OTUs’ relative abundances, linear discriminant analysis (LDA) was performed using the online interface Galaxy (http://huttenhower.sph.harvard.edu/lefse/) with an alpha value < 0.05 and a LDA score > 3 [46, 47]. Only those OTUs with relative abundances higher than 0.5% were selected for subsequent analyses [48, 49].

To assign the phylogenetic position of specific fungal taxa, we used the taxonomic classification provided by the UNITE database and built de novo phylogenetic trees using ‘best match’ reference sequences obtained from the NCBI database. The identities of these taxa were further validated by primer-specific quantitative polymerase chain reaction (qPCR) assays and the isolation in culture media of these targeted taxa [50].

### Isolation and identification of *Foc*TR4 pathogen in the experimental area

To validate the presence of *Foc*TR4 in our samples, diseased bananas were collected from our experiment site at Ledong County. These materials were subjected to *Fusarium* sp. isolation and screening as previously reported [30, 34]. The DNA of the obtained isolates and one pathogenic *Foc* strain stored in our lab [51] was extracted and subjected to a preliminary identification using the sequencing and analysis of the ITS region. Next, the gene-specific primer sets *Foc*TR4-F/*Foc*TR4-R and EF1-F/EF2-R or VCG01213-16F1/VCG01213-16R2 and EF1-F/EF2-R were used to conduct two multiplex-PCR analyses to identify the pathogen type at the molecular level. For additional details of primers, see Table S3.

### Quantification of *Foc*TR4, *A. fumigatus*, and *F. solani* abundances

Primer-specific qPCR systems were used to quantify the absolute abundances (target gene copies g^−1^ dry soil) of the pathogen *F. oxysporum* f. sp. *cubense* tropical race 4 (*Foc*TR4), and the antagonistic fungal taxa *A. fumigatus*, and *F. solani* in all soil samples. For that, we used the primer sets *Foc*Sc-1/*Foc*Sc-2 [52], AfumiF1/AfumiR1 and the probe AfumiP1 [53], Fs-F/Fs-R and the probe 2 [54], respectively. Each standard curve was established by 10-fold serial dilutions of plasmids containing either *Foc*TR4, *F. solani*, or *A. fumigatus* targeted sequences (see Table S3 for additional details). The qPCR amplifications contained 2 μl of the target DNA, 10 μl of Synergy Brands (SYBR®) Green premix Ex Taq™ (2×), 0.4 μl of each primer, 0.4 μl of ROX Reference Dye II, and 7.2 μl of nuclease-free and sterile water. Each assay was performed in triplicate, and the results are expressed as log_10_ values (i.e., target gene copies g^−1^ soil) prior to statistical analysis.

### Isolation and identification of antagonistic taxa, and *Foc*TR4 inhibition experiments

Culturable fungi were isolated from soils using the Rose Bengal Agar (RBA) (Hopebio Company, Qingdao, China) and the Potato Dextrose Agar (PDA) media (Hopebio Company, Qingdao, China), supplemented with 25 mg ml^−1^ of chloramphenicol to inhibit bacterial growth. A total of 20 g well-mixed soil of each treatment was added to 180 ml of sterile water (10^−1^ soil dilution) and serially diluted to 10^−2^, 10^−3^, and 10^−4^ [55]. Next, 0.1 ml of the soil solution of each concentration was plated in both media. Each dilution per treatment was performed with three replicates, and all petri dishes were incubated at 28 °C. After 3 days, fungal colonies were counted and numbered up to the point the number of fungal colonies reached stable counts. A total of 30 colonies were randomly selected per treatment, resulting in a total of 150 fungal isolates. These fungal isolates were purified by replication, and their respective DNAs were extracted and subjected to amplification using the primer set ITS1/ITS4 (Table S3). These fragments were amplified and sequenced by the TSINGKE Biological Technology Company (Beijing, China), and individual sequences were compared against the NCBI GenBank database using blast [56]. Based on the outcome of this analysis, three *A. fumigatus* and three *F. solani* isolates were selected for follow-up experiments. Worth mentioning, these isolates displayed high ITS nucleotide similarities with sequences obtained from the community high-throughput sequencing.

The three *A. fumigatus* and three *F. solani* isolates were tested for their potential to inhibit the growth of the pathogen *Foc* (*Foc*TR4), which was isolated and stored in our lab [51]. These isolates were tested using two experimental assays. A dual culture experiment consisted of the inoculation of each individual strain against the pathogen *Foc*TR4 using the PDA medium [57]. The inoculation was performed by transferring 3 mm (diameter) of third-day colonies of each fungus on opposite sides of the petri dish. The volatile-mediated inhibition experiment consisted of inoculating each fungal isolate and the pathogen *Foc*TR4 at the center of individual petri dishes. Then plates containing the pathogen were inverted on top of individual plates containing the isolates, sealed, and incubated in a thermostatic water-jacket at 28 °C [58]. Each treatment combination had three independent replicates, and colony diameters were measured in both assays after 3 days.

### Plant residue extracts and their effects on the growth of *Foc*TR4, *F. solani*, and *A. fumigatus*

A total of 60 g (dry weight) of each plant residue powder was added to 540 ml of carbinol and shook for 24 h at 4 °C. Then, the obtained solution was filtered using a 0.22-μm organic filter membrane [59, 60]. The filtered solution was dried using a rotatory evaporation system, and the concentrated substances were dissolved in 60 ml of sterile deionized water. A volume of 6 ml of a solution derived from each plant residue type was added to 294 ml of PDA medium (i.e., 2% concentration, volume ratio: V/V) after sterilization. The four treatments used were the following: above-ground banana residue extract (BSE), below-ground banana residue extract (BRE), above-ground pineapple residue extract (PSE), below-ground pineapple residue extract (PRE), and control using sterile deionized water (CKE). As similarly described above, 3 mm diameter colonies of *Foc*TR4, *A. fumigatus*, or *F. solani* were inoculated on the center of each plate and the growth diameters were measured after 3 days of incubation at 28 °C. Specifically, for the pathogen *Foc*TR4, the potential effects of higher amounts of the residue extracts on growth were tested, including 2%, 5%, 10% volume ratios (V/V). This was carried out to closely approximate differences in concentrations that could be observed in field settings. To account for potential effects of nutrient differences in the different extracts influencing fungal growth, all extracts were subjected to measurements of TC, TN, TK, and TP, as performed by Bao [36].

### Metabolic profiling of *Foc*TR4 and antagonistic fungal taxa

Biolog FF MicroPlate™ (Biolog Company, USA) were used to obtain the metabolic profile of *Foc*TR4 in comparison to isolates of *A. fumigatus*, *F. solani*, three other species of *Fusarium* spp. that occurred at high abundances during fungal isolation in the treatment containing banana residues (Table S4), and three other species of *Aspergillus* spp. known to have no antagonistic effect on *Foc*TR4. Each fungal strain was cultured on PDA medium to obtain spores. A 0.1 ml of spore suspension of each strain (adjusted to 75% ± 2% with a turbidity meter in FF inoculation fluid, FF-IF) was inoculated into each well in a Biolog FF MicroPlate™. Triplicated MicroPlates per treatment were placed in an aerobic Omnilog incubator reader (Biolog Inc., USA) at 20 °C (i.e., the optimal growth temperature as recommended by the Westerdijk Fungal Biodiversity Institute culture collection). Colorimetric values at 590 nm were measured at 12 h, 24 h, 36 h, 48 h, 60 h, 72 h, 96 h, 120 h, 144 h, and 168 h, and blanked against the control wells. Results were considered positive only when differences between the first and last days were observed across all replicates. The percentage of common carbon source utilization between each strain and *Foc*TR4 was obtained by (*A*∩*B*)/B*100%, where *A* is the available carbon source of antagonistic taxa and *B* is the available carbon source of *Foc*TR4.

### Validation experiment of disease suppression by *A. fumigatus* and *F. solani*

A total of three *F. solani* (F.S-11, F.S-17, and F.S-18) and three *A. fumigatus* (A.F-7, A.F-12, and A.F-15) isolates were used in the validation pot experiment against *Foc*TR4. A culture of *Escherichia coli* (EC) and one sterile water treatment (CKW) were used as negative controls. Microbial spores or cells were inoculated into the soil at a final concentration of 10^4^ colony forming unit (CFU) g^−1^ dry soil, and each pot was transplanted with one banana seedling. After 45 days, soil samples from each of the 6 pots per treatment were collected and the abundance of *Foc*TR4 was determined via qPCR (described above). This entire experiment was repeated twice to test for the consistency and reproducibility of the results.

### Statistical analyses

Data obtained from the greenhouse experiment were analyzed using linear models based on stepwise selection (step () function in R) to obtain main explanatory variables. In order to get accurate results, the Akaike Information Criteria (AIC) and Bayesian Information Criterion (BIC) were utilized at the same time [61]. Structural equation model was used to visualize the potential direct and indirect effects of pathogen density on disease incidence in the pot experiment, this analysis was carried out using the R package *sem* [62]. Boxplots were generated using the R package of *ggpolt2* [63]. Correlation analyses were conducted with the R packages *ggpolt2*, *reshape2*, and *psych*, and the *P* values were adjusted for false discovery rate (FDR) [63–65]. Histograms and curve charts were plotted using the R packages *ggpolt2* and *Rmisc* [63, 66]. Tukey’s HSD tests (*P* < 0.05) were performed using the SPSS 22.0 software (IBM, USA). Non-normal data were square-root or log-transformed to improve normality and homoscedasticity for statistical analysis. The software Molecular Evolutionary Genetics Analysis Version 7.0 (MEGA7) was used for phylogenetic analysis of sequences obtained from fungal isolates [67]. The final alignment included the 20 best matches of strains downloaded from NCBI database.

## Results

### *Fusarium* wilt disease incidence and *Foc*TR4 abundance

**Fig. 1:**
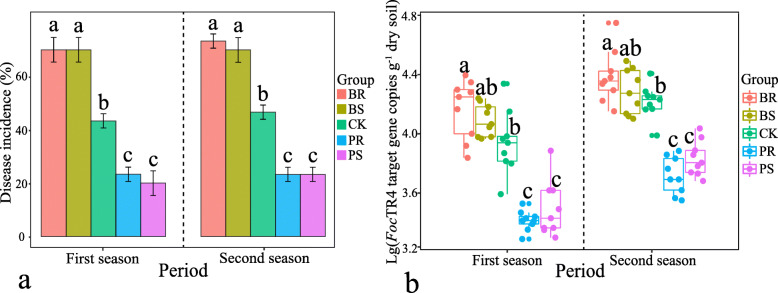
**a** Bar chart displaying the incidence (in %) of the *Fusarium* wilt disease in each treatment at two independent experiments (indicated as first and second seasons). **b** Copy numbers of *Foc*TR4 target gene copies (qPCR) per gram of dry weight soil. Data are shown per each treatment at two independent experiments (indicated as first and second seasons). BS above-ground banana residue; BR, below-ground banana residue; PS, above-ground pineapple residue; PR, below-ground pineapple residue; and CK, control treatment without residue. Different lowercase letters indicate statistically significant differences (*P* < 0.05) according to Tukey’s HSD test

Compared with the CK, the treatments amended with either pineapple above-ground (PS) or below-ground (PR) residues had significant (*P* < 0.05) negative effects on the occurrence of the banana *Fusarium* wilt disease. On the other hand, the treatments amended with banana above-ground (BS) or below-ground (BR) residues had a significant (*P* < 0.05) positive effect on the disease incidence compared to CK (Fig. 1a). Worth mentioning, no statistical differences were detected between treatments with positive effects (PS and PR) and between treatments with negative effects (BS and BR), both in terms of disease incidence and *Foc*TR4 copy numbers (Fig. 1a, b). Besides, the disease symptoms in treatments containing banana residues and in the CK were more severe than that observed in treatments containing pineapple residues. These symptoms showed a similar trend with the banana disease incidence (Table S5). Moreover, these results were found to be reproducible at two independent experiments (Fig. 1). The copy number of *Foc*TR4 per gram of soil was significantly correlated with the wilt disease incidence (Fig. S1, first season: *r* = 0.83, *P*(FDR) < 0.001; second season: *r* = 0.93, *P*(FDR) < 0.001).

### Differences in fungal communities profiling across treatments

**Fig. 2:**
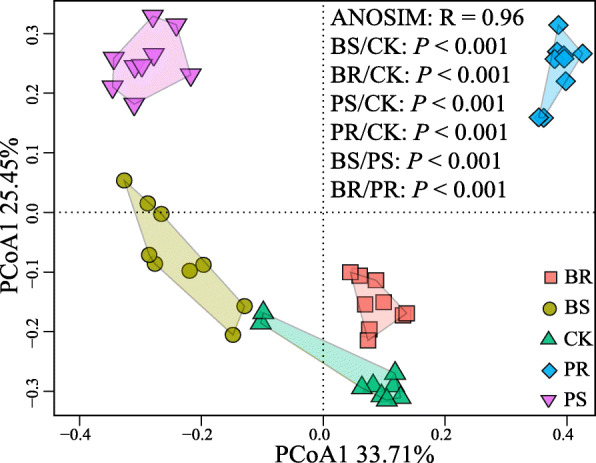
Principal coordinate analysis (PCoA) of soil fungal communities based on the Bray-Curtis distances. BS, above-ground banana residue; BR, below-ground banana residue; PS, above-ground pineapple residue; PR, below-ground pineapple residue; and CK, control treatment without residue; ANOSIM, analysis of similarities

As visualized in the PCoA plot, different crop residues exerted significant influences on the soil fungal communities, and treatments were all significantly different from one another based on pairwise comparisons (ANOSIM: *r* = 0.96, *P* < 0.001) (Fig. 2a). Interestingly, the first axis of the PCoA explaining 33.71% of the variation segregated the treatments containing below-ground plant residues (BS and PS) from the treatments containing above-ground plant residues (BR and PR). The second axis explaining 25.45% of the total variation segregated the treatments according to their respective plant identities (PS and PR–BS and BR) (Fig. 2a). The control treatment (CK) clustered close to the treatments BS and BR, albeit being statistically different. This indicates a relatively lower influence of banana residues on the fungal community structure compared to pineapple residues. Worth mentioning, the bacterial communities across all treatments were also profiled (additional details can be seen in Supplementary Information), but no significant differences (ANOSIM: *P* > 0.1) were found (Fig. 2a). Moreover, the copy numbers of *Foc*TR4 target gene (qPCR) were found to be significantly correlated with the disease incidence and to be related to the overall composition of soil fungal communities (Fig. S2b). Worth mentioning, there was no significant effect of residue amendment on soil physicochemical properties that explain differences in fungal communities across treatments (Fig. S2c). Taken together, these results suggested the higher importance of fungal communities rather than that of bacteria on *Fusarium* wilt disease incidence and suppression in our experimental system.

### Identification of fungal taxa potentially involved in *Fusarium* wilt disease suppression

**Fig. 3:**
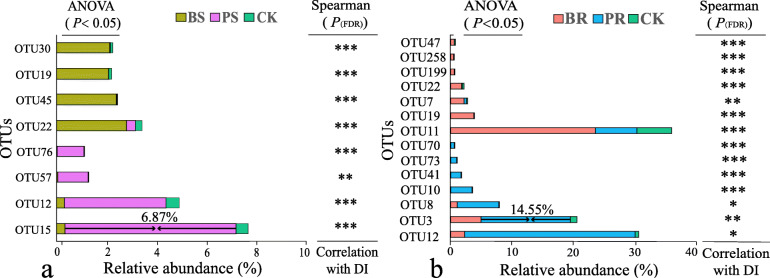
Differential abundance and correlation analyses of fungal OTUs that significantly (*P* < 0.05) related to the *Fusarium* wilt disease incidence. **a** Analysis including below-ground crop residues and the control treatment. **b** Analysis including above-ground crop residues and the control treatment. Correlation analysis was based on Spearman and obtained *P* values were corrected using false discovery rate (FDR). *P < 0.05, **P < 0.01, and ***P < 0.001. DI, Banana *Fusarium* wilt disease incidence; BS, above-ground banana residue; BR, below-ground banana residue; PS, above-ground pineapple residue; PR, below-ground pineapple residue; and CK, control treatment without residue

Several OTUs were found to have statistically different elative abundances across treatments based on LDA analysis. The analysis was carried out to partition OTUs that significantly increased in PR compared to BR and CK or in PS compared to BS and CK (Fig. S3a and Fig. S3b). We also tested the extent to which these OTUs were negatively correlated with the disease incidence (Fig. 3a, b). Then, the contribution of these OTUs to disease suppression was evaluated based on linear models. The residuals of the two models were in accordance with the normal distribution (Shapiro-Wilk test, *P* > 0.05), and the majority of the differences in disease incidence were explained (*R*^2^ > 0.88, *P* < 0.001, proportion of variance explained > 89%). Each variance inflation factor (VIF) value of the final key taxa was < 5, thus collectively indicating the validity and strength of the linear models. Specifically, OTU3 (*F. solani*) and OTU15 (*A. fumigatus*), which were with relative abundances of 14.55% in PR and 6.87% in PS, respectively, were the top two OTUs that most significantly related to the disease suppression (Table 1). In contrast, some OTUs were also identified to significantly correlated with the increase in the disease incidence for the treatments BR and BS (analysis of variance: ANOVA, *P* < 0.05; Spearman, *P*_(FDR)_ < 0.05; see Fig. 3a and Fig. 3b for details). Besides, the OTU22 (*Humicola* sp.) and OTU11 (unclassified Ascomycota) were the top 2 OTUs that most significantly related to disease incidence (Table 1). See Fig. S4 and Table S6 for additional details.

**Table 1:** Linear models displaying the relationship between specific OTUs and the incidence or suppression of *Fusarium* wilt disease according to either above-ground or below-ground residue incorporation

Key taxa	Disease incidence (above-ground residues)				Key taxa	Disease incidence (Below-ground residues)			
	*P*	VIF	*r*	Relative Importance		*P*	VIF	*r*	Relative importance
OTU15	*P* < 0.001***	1.21	− 0.78	65.73%	OTU3	*P* < 0.001***	1.05	− 0.58	37.60%
OTU22	*P* < 0.001***	1.21	0.30	23.40%	OTU11	*P* < 0.001***	1.85	0.35	23.11%
					OTU22	*P* < 0.001***	1.92	0.39	29.71%
Model summary	*R*^2^_-adj_ = 0.88, *P* < 0.001				Model summary	*R*^2^_-adj_ = 0.89, *P* < 0.001			
Shapiro-Wilk normality test	*W* = 0.94, *P* > 0.05				Shapiro-Wilk normality test	*W* = 0.98, *P* > 0.05			
Proportion of variance explained by model: 89.13%					Proportion of variance explained by model: 90.42%				

Notes: the model summary displays *P* values lower than 0.05 (ANOVA), *R*^*2*^*-adj* adjusted *R*^2^ values, *VIF* variance inflation factor, *r* standardized coefficients

### Quantification of potentially suppressive fungal taxa in soil and SEM analyses

**Fig. 4:**
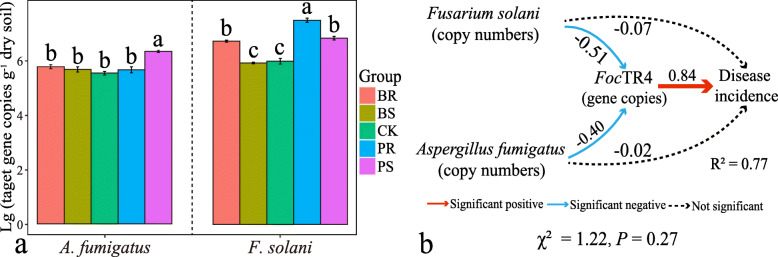
**a** Bar charts displaying the absolute abundances (target gene copy numbers) of *A. fumigatus* and *F. solani* in the soil. **b** Structural equation model (SEM) linking the abundances of *A. fumigatus*, *F. solani*, and *Foc*TR4, and their relationships with the disease incidence. *R*^2^, *χ*^2^, and *P* values denote the fit of the model. BS, above-ground banana residue; BR, below-ground banana residue; PS, above-ground pineapple residue; PR, below-ground pineapple residue; and CK, control treatment without residue. Different lowercase letters indicate statistically significant differences (*P* < 0.05) according to Tukey’s HSD test

The values of absolute quantification (target gene copies per gram of soil) of the fungal taxa *F. solani* and *A. fumigatus* across all treatments were combined with the absolute quantification of *Foc*TR4 target gene copies to model the disease incidence using structural equation modeling (SEM). Both potentially suppressive taxa (*F. solani* and *A. fumigatus*) were found to have a direct negative effect on *Foc*TR4 copy numbers (*ρ* = **−** 0.51 and *ρ* = **−** 0.40, respectively). As expected, the absolute values of *Foc*TR4 copies have a direct relationship with the disease incidence (*ρ* = 0.84) (Fig. 4b). In addition, there were significant correlations between the target gene copies numbers (absolute quantification) of these taxa and their respective relative abundances obtained by high-throughput sequencing (*P* < 0.05, Fig. S5a).

### Quantification of potentially suppressive fungal taxa and pathogen in plant residues

**Fig. 5:**
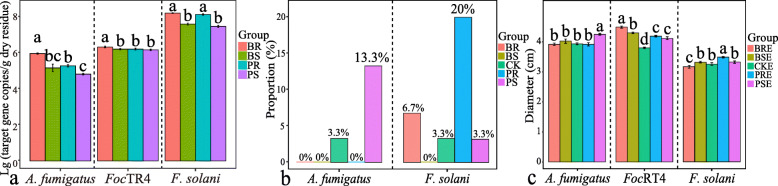
**a** Absolute quantification (target gene copies) of *A. fumigatus*, *F. solani*, and *Foc*TR4 in each crop residue. **b** Proportion of culturable *A. fumigatus* and *F. solani* obtained in each treatment (*n* of individual taxa/30 isolates per treatment). **c** Growth diameters of *A. fumigatus*, *F. solani*, and *Foc*TR4 on PDA medium supplemented with different plant residue extracts. BSE, above-ground banana residue extract; BRE, below-ground banana residue extract; PSE, above-ground pineapple residue extract; PRE, below-ground pineapple residue extract; and CKE, control using sterile deionized water. BS, above-ground banana residue; BR, below-ground banana residue; PS, above-ground pineapple residue; PR, below-ground pineapple residue; and CK, control treatment without residue. Different lowercase letters indicate statistically significant differences (*P*< 0.05) according to Tukey’s HSD test

No significant difference in the abundances of *A. fumigatus* between BS and PS, and in the abundances of *F. solani* between BR and PR were found (*P* > 0.05) (Fig. 5a). The abundance of *Foc*TR4 in BR was significantly higher than that observed in other crop residues (*P* < 0.05) (Fig. 5a). Also, no positive correlations between the target gene copy numbers in the soils and the target gene copy numbers in the residues for these potentially suppressive taxa and the pathogen was observed (*r* < 0, Fig. S5b). This likely indicates the abundances of these taxa not relate to the residue incorporation in the soil.

### Isolation and identification of culturable fungal strains in the soil

A total of 26 isolates taxonomically affiliated with *Fusarium* spp. were obtained from diseased banana plants collected in our long-term experimental site. By using a combination of morphological analysis with sequencing data, 22 strains were identified as *Fusarium oxysporum* (Table S7). Two multiplex-PCR systems validated that all these 22 strains and the pathogenic *Foc* strain in our lab were *Foc*TR4. See Fig. S6 for details. In addition, a total of 18 distinct fungal genera were identified in harvest soil based on isolation and ITS sequence analysis. Isolates taxonomically affiliated with *A. fumigatus* and *F. solani* were found at the highest proportion in the soils obtained from the PR and PS treatments (Fig. 5b).

### Inhibition experiment of *Foc*TR4 and metabolic profiles of *F. solani*, *A. fumigatus*, and *Foc*TR4

Overall, *F. solani* isolates had no antagonism effect on *Foc*TR4 growth (Fig. S7a, Fig. S7b). However, *A. fumigatus* isolates displayed clear antagonistic effects via the potential secretion of secondary metabolites and the production of growth-inhibiting volatiles (Fig. S7c, Fig. S7d, and Fig. S7e). The metabolic profile of these fungi showed all isolates to reach a stable growth condition after 96 h. *F. solani* isolates had a greater overlap in terms of carbon utilization with the pathogen *Foc*TR4, compared to *Aspergillus* spp. isolates and other *Fusarium* spp. isolates (Fig. S8a and Fig. S8b). The values of the average well color of overlapping carbon sources between *Foc*TR4 and *F. solani* displayed no significant differences and were higher than that of *Aspergillus* spp. and *Fusarium* spp. isolates (Fig. S8c). Taken together, these results support a higher metabolic similarity (in terms of carbon source utilization) between *Foc*TR4 and *F. solani* (about 96.3%), than between *Foc*TR4 and *A. fumigatus* (about 64.61%) (Fig. S8d).

### Effects of plant residue extracts on the growth of *F. solani*, *A. fumigatus,* and *Foc*TR4

The addition of both above-ground and below-ground pineapple residue extracts (PSE and PRE) had a positive effect on the growth of *F. solani* and *A. fumigatus* (Fig. 5c). In addition, no significant effects were found on the colony diameters of *Foc*TR4, *F. solani*, and *A. fumigatus* and the total nutrient content present in each residue extract (Fig. S9). Interestingly, all residue extracts exerted a positive effect on the colony growth of *Foc*TR4, even when the concentration of the extracts ranged from 2 to 10%. These positive effects on pathogen growth were found to be pronounced in treatments containing banana residue extracts (BSE and BRE) (Fig. S10a and Fig. S10b). For a detailed information of total nutrient contents in each plant residue extracts, see (Fig. S10c).

### Validation of disease suppression mediated by *F. solani* and *A. fumigatus* isolates

**Fig. 6:**
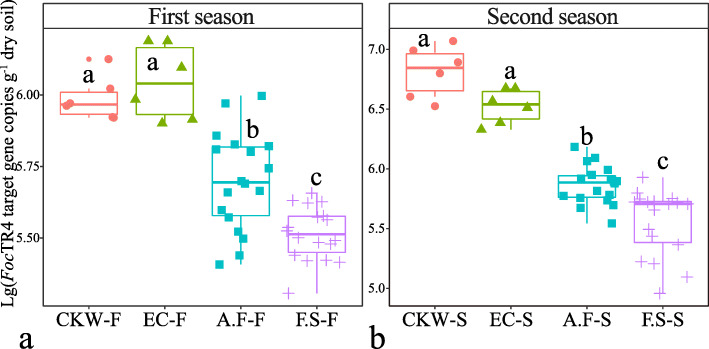
Absolute abundance of *Foc*TR4 target gene copies in soil across different inoculation treatments. **a** First season and **b** second season (two independent experiments). CKW-F, control inoculated with sterile water; EC-F, control inoculated with *Escherichia coli*; A.F-F, inoculation with a combination of three *A. fumigatus* isolates (A.F-7, A.F-12, and A.F-15); F.S-F, inoculation with a combination of three *F. solani* isolates (F.S-11, F.S-17, and F.S-18). Different lowercase letters indicate statistically significant differences (*P* < 0.05) according to Tukey’s HSD test

The two independent pot inoculation experiments revealed the three isolates of *F. solani* and *A. fumigatus* to exert significant effects on the control of the pathogen density in the soil. The addition of these taxa significantly and negatively affected the *Foc*TR4 target gene copy numbers in the soils (*P* < 0.05). The treatment inoculated with *F. solani* isolates had a stronger negative effect than the treatment inoculated with *A. fumigatu*s isolates, and no significant differences were observed between the two control treatments (Fig. 6a). The second independent experiment showed a similar trend in our results, thus supporting the consistency and reproducibility of our findings (Fig. 6b). For additional details of each strain, see Fig. S11a and Fig. S11b.

## Discussion

There is still a paucity of information on the extent to which the incorporation of different plant residues in soils affects multiple beneficial functions, such as ecological interactions between organismal types and the establishment of disease-suppressive systems [1, 27]. Here, we studied an important soil-borne pathogen (*Foc*TR4) infecting banana plants to show that the management of distinct plant residues in the soil results in a direct status of either disease incidence or suppression. The experimental set-up consisted of different types (above- and belowground materials) of two plant species (banana and pineapple). This design allowed for a fine partitioning of the effects of residue incorporation differentially modulating changes in the soil microbiome. Importantly, as we initially observed that only minor changes occur across treatments in bacterial communities in the soil, a major focus of our study was set on investigating the role of potentially suppressive fungal taxa that emerged in our system.

The incorporation of both above- and below-ground pineapple residues (PR and PS) into the soil was found to significantly decrease the pathogen density in the soil and the incidence of the *Fusarium* wilt disease. This finding aligns with our previous studies showing that the use of a pineapple-banana crop rotation system can effectively minimize the incidence of this pathogen [68]. On the other hand, the incorporation of banana residues into the soil was found to have an opposite effect, thus increasing the disease incidence. As such, the incorporation of susceptible host substrate (banana residues) into the soil under a monoculture system, in this case, results in negative plant-soil feedback, i.e., by promoting the continuous selection of the pathogen in the system negatively impacting crop performance [69, 70]. On the contrary, the introduction of pineapple residues results in positive plant-soil feedback, i.e., via lowering the pathogen pressure by enriching beneficial antagonistic taxa that promote plant disease suppression [71].

The use of either above- or below-ground residues from either banana or pineapple plants differentially influenced the structure of fungal soil communities. This result corroborates with previous findings showing that the soil microbiome can be dynamically impacted by different types and concentrations of organic amendments [72]. In our study system, these changes in fungal communities resulted either in conducive (banana residues) or suppressive (pineapple residues) soil systems [73, 74]. Specifically, in our experiment, this suppressive status emerged as a function of changes in the soil fungal communities, rather than mediated by changes in soil physicochemical properties. This was also further corroborated in the structural equation modeling (SEM) that traced the relationships among microbial communities, pathogen density, and the banana disease incidence.

The incorporation of pineapple residues (PS and PR) was found to significantly increase the relative abundances of *F. solani* (OTU3) and *A. fumigatus* (OTU15). We later confirmed that this trend in relative abundance aligns with their absolute abundances in these soil treatments. By including their abundances in an SEM, both taxa were found to negatively correlate with the density of the pathogen in soil, which directly affect the disease incidence in the model. Based on the results of screening experiments, the relative abundances of *A. fumigatus* and *F. solani* show similar tendency with their proportion in all culturable fungi. Inspired by these findings, we performed co-culture experiments to test the in vitro potential of isolates belonging to these taxa in controlling the colony growth of *Foc*TR4. For that, we also surveyed our system to provide further confirmation that *Foc*TR4 is indeed the major causal agent of the *Fusarium* wilt disease in the area of our study, as previously reported [34].

Co-culture assays that combined the pathogen inoculation with a series of potentially suppressive isolates of *A. fumigatus* were able to validate the in vitro suppressive capacity of these taxa via the secretion of inhibitory compounds and/or via the production of volatile compounds. In addition, we were able to determine that pineapple residue extracts are in fact able to stimulate the growth of *A. fumigatus* and *F. solani*, even though there was no significant correlation between the colony diameters and total nutrient contents in these extracts (i.e., TP, TK, TC, and TN). These results indicate that perhaps the presence of specific substances in the residues would be beneficial in promoting the growth of these suppressive taxa [75, 76]. Likewise, the banana residue extracts were also found to promote the growth of *Foc*TR4, which corroborate previous findings [77]. It is also worth noting that the pineapple residue extracts had also a positive effect on the growth of *Foc*TR4. This result opposes previous findings showing that residues are able to promote disease suppression tend to exert a negative effect on pathogen growth [78]. In our study, these positive pathogen-promoting effects may reduce directly by the growth stimulation of suppressive taxa in the system that can outcompete the pathogen. The metabolic profiling of *A. fumigatus* and *F. solani* and their range of overlap with that of the pathogen *Foc*TR4 provides further support to this interpretation. Thus, particularly in our study, we speculate that the substances in pineapple residues cannot decrease the banana disease incidence by directly inhibiting the number of pathogens in the soil, but it may happen indirectly by inducing the population of antagonists.

The pathogen-inhibiting capacity of *A. fumigatus* was further confirmed in a pot experiment, and a direct pathogen-inhibiting effect was observed in vitro. *A. fumigatus* has already been reported to secrete antifungal compounds able to inhibit pathogens, including *Fusarium* sp. [79, 80]. On the other hand, isolates affiliated with *F. solani* were also found as a potential suppressive taxon in our system. Interestingly, strains belonging to the species *F. solani* are well-known pathogens causing diseases in different crops, such as chili and eggplants [81, 82]. Despite we did not find significant effects of *F. solani* in inhibiting the growth of the pathogen *Foc*TR4 in vitro, the inoculation of *F. solani* in two independent experiments showed reproducible results on the suppressive potential of this taxon. This represents a novel finding, as no previous report on the suppressive potential of *F. solani* against *Foc*TR4 yet exists in the literature. It is worth noting that *F. solani* and *Foc*TR4 belong to the same genus, *Fusarium*. As indicated above, these taxa have very similar metabolic profiles in terms of carbon source utilization (i.e., 96.3%). This likely indicates a more similar niche preference of these taxa compared to other microbes enriched in our system [83]. As such, is it expected that competition for resources between these taxa might be the major mechanism involved in the pathogen suppression [84]. Taken together, both taxa *A. fumigatus* and *F. solani* can act on the suppression of the pathogen *Foc*TR4 via two distinct mechanisms, i.e., the production of antagonistic compounds and the ecological competition for nutrients in the system, respectively.

## Conclusions

**Fig. 7:**
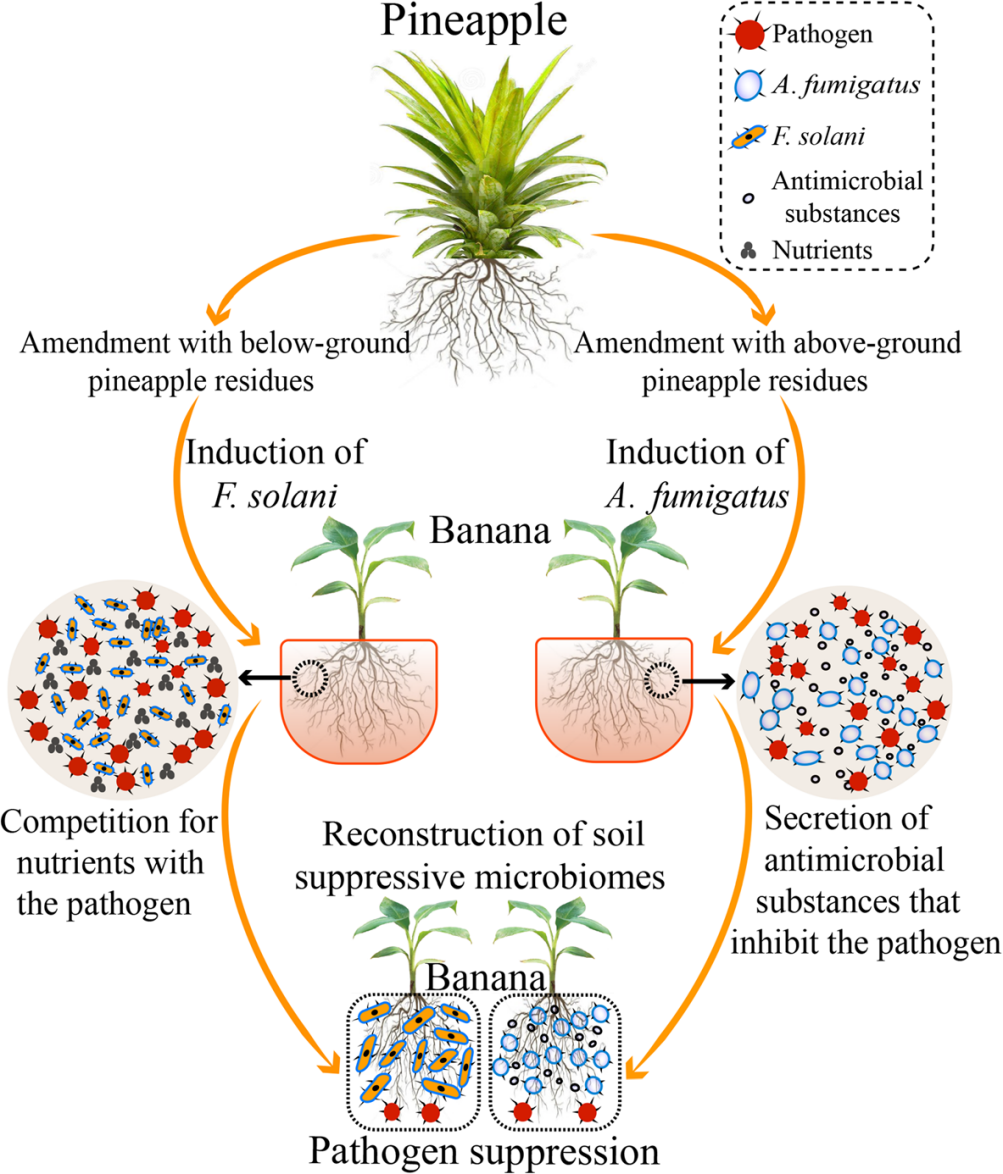
Conceptual model displaying the potential role of suppressive fungal taxa influencing the pathogen density (qPCR) in the soil and the occurrence of *Fusarium* wilt disease

Previous studies in the literature have been suggesting that agricultural practices can be optimized to recover or strengthen desirable functions in soils by inducing changes in the resident microbiome [7]. Our study contributes to this body of research by showing that the incorporation of specific plant residues in the soil can trigger soil suppression against an important soil-borne pathogen (*Foc*TR4). Here, we depict this suppressive status to be modulated by trackable changes in the soil fungal communities, and highlight two fungal taxa potentially directly involved in the pathogen suppression. We further provide pieces of evidence for their suggested distinct and complementary modes of action, and later validate their suppressive potential under well-controlled pot inoculation experiments (Fig. 7). Taken together, our study may provide new avenues for the exploration of agricultural practices focused on beneficial outcomes that directly impact soil health and crop productivity in a viable and sustainable manner.

## Supplementary Information

**Additional file 1: **Table S1. Nutrient content (g/kg) present in the pineapple and banana residues. Table S2. Nutrient content corrections (g/pot) carried out across treatments. Table S3. Primers and probe sequences used in this study. Table S4. Proportion of culturable *Fusarium* spp. obtained in each treatment. Table S5. *Fusarium* wilt disease severity and symptoms across treatments. Table S6. Taxonomic identification of fungal OTUs. Table S7. *Fusarium* spp. isolation and strain level identification. Fig. S1 Correlation analysis between *Foc*TR4 target gene copies and the *Fusarium* wilt disease incidence in two independent experiments (indicated as first and second seasons. Correlation analyses were based on Spearman and the *P*-values were corrected for False Discovery Rate (FDR). Fig. S2 a Principal coordinate analysis (PCoA) of soil bacterial communities based on the Bray-Curtis distances. BS: above-ground banana residue, BR: below-ground banana residue, PS: above-ground pineapple residue, PR: below-ground pineapple residue, and CK: control treatment without residue. ANOSIM: analysis of similarities. b Structural equation model (SEM) linking the microbial (bacterial and fungal) community compositions (based on PCoA), the pathogen *Foc*TR4 density (copy numbers, qPCR), and their respective relationships with the disease incidence. R^2^, χ2, and P-values denote the fit of the model. c Redundancy analysis (RDA). “***” represents the *P* < 0.001. AP: rapid available phosphorus, AK: soil available kalium, NO_3_^-^: nitrate nitrogen, NH_4_^+^: ammonium nitrogen, pH: pH value, EC: electrical conductivity, TOC: total organic carbon, DI: disease incidence, *Foc*RT4: *Fusarium oxysporum* f. sp. *cubense* tropical race 4. Fig. S3 Linear discriminant analysis (LDA). a Analysis including below-ground crop residues and the control treatment, and b analysis including above-ground crop residues and the control treatment. BS: above-ground banana residue, BR: below-ground banana residue, PS: above-ground pineapple residue, PR: below-ground pineapple residue, and CK: control treatment without residue. Fig. S4 Phylogenetic reconstructions of a *F. solani* isolates (FS11, FS17, and FS18) and OTU3, b *A. fumigatus* isolates (AS-7, AS-12, and AS-15) and OTU15, and c OTU11. Each independent phylogenetic reconstruction included best match sequences obtained from the NCBI database for taxonomical inferences. Fig. S5 a Correlation plots displaying the relationships between the target gene copy numbers (qPCR) of specific fungal taxa (*A. fumigatus* and *F. solani*) in soil and their respective relative abundances in soil obtained by Illumina Miseq sequencing. b Correlation plots displaying the relationships between the target gene copy numbers (qPCR) of specific fungal taxa in crop residues (*A. fumigatus* and *F. solani*) and the target gene copy numbers (qPCR) of specific fungal taxa in soil. Correlation analyses were based on Spearman and the *P*-values were corrected for False Discovery Rate (FDR). Fig. S6 a Results from the multiplex-PCR system based on the targets TEF-1α (650bp, positive control) and *Foc*TR4 (463bp). b Results from the multiplex-PCR system based on the targets TEF-1α (650bp, positive control) and *Foc*TR4 (VCG 01213/16) (455bp). *F.s*: *F. solani* (negative control). Fig. S7 Results obtained from the co-culture experiments to test the potential of *F. solani* and *A. fumigatus* isolates in inhibiting the colony growth of the pathogen *Foc*TR4. a, b Display the potential effects on *Foc*TR4 colony growth mediated by volatile compounds and secreted substances produced by *F. solani*. c, d Display the potential inhibiting effects on *Foc*TR4 colony growth mediated by volatile compounds and secreted substances produced by *A. fumigatus*. e Antagonistic effects of sterile fermentation fluid from *A. fumigatus* on *Foc*RT4, CK: added water, W: added fluid medium, A.F (7, 12,15): added sterile fermentation fluid from *A. fumigatus*. Fig. S8 a Number of carbon source utilization by each individual fungal taxa thought time. b Carbon source metabolic rate of each fungal taxa indicated by the average well color development (AWCD). c Average well color (AWC) values of common carbon source utilization between *Foc*TR4 and each individual fungal taxa tested at the 96 hours time point. d Percentage of common carbon source utilization between *Foc*TR4 and each individual fungal taxa tested at the 96 hours time point. Different lowercase letters indicate statistically significant differences (*P* < 0.05) according to Tukey's HSD test. Fig. S9 Correlation analyses between the colony growth (in diameter) of each specific fungal taxa and the nutrient contents (mg/kg) in the residue extracts. a Pathogen *Foc*TR4, b *A. fumigatus*, and c *F. solani*. TN: total nitrogen, TC: total carbon, TP: total phosphorus, TK: total kalium. Correlation analyses were based on Spearman and the *P*-values were corrected for False Discovery Rate (FDR). Fig. S10 Visualization of the colony growth of the pathogen *Foc*TR4 on media supplemented with 2% (a) and 2%, 5% and 10% (b) of plant reside extracts (BSE, BRE, PSE, PRE). c Nutrient contents (mg/kg) in each crop reside extract. BSE: above-ground banana residue extract, BRE: below-ground banana residue extract, PSE: above-ground pineapple residue extract, PRE: below-ground pineapple residue extract, CKE: control using sterile deionized water. TN: total nitrogen, TC: total carbon, TP: total phosphorus, TK: total kalium. Different lowercase letters indicate statistically significant differences (*P* < 0.05) according to Tukey's HSD test. Fig. S11 Absolute abundance of *Foc*TR4 target gene copies in soil across different inoculation treatments. a first season and b second season (two independent experiments). CKW-F: control inoculated with sterile water, EC-F: control inoculated with *Escherichia coli*, A.F-F: inoculation with a combination of three *A. fumigatus* isolates (A.F-7, A.F-12, and A.F-15), F.S-F: inoculation with a combination of three *F. solani* isolates (F.S-11, F.S-17, and F.S-18). Different lowercase letters indicate statistically significant differences (*P* < 0.05) according to Tukey's HSD test.

## Data Availability

All raw sequence data have been made available in the NCBI Sequence Read Archive (SRA) database under the accession number PRJNA670608.
