# Supplementary material for: Development of fungal-mediated soil suppressiveness against Fusarium wilt disease via plant residue manipulation
Source: Microbiome. 2021 Oct 12;9:200. doi: 10.1186/s40168-021-01133-7 (PMC8507339; doi:10.1186/s40168-021-01133-7)
Supplement: Supplementary file 2 — Additional file 1: Table S1. Nutrient content (g/kg) present in the pineapple and banana residues. Table S2. Nutrient content corrections (g/pot) carried out across treatments. Table S3. Primers and probe sequences used in this study. Table S4. Proportion of culturable Fusarium spp. obtained in each treatment. Table S5. Fusarium wilt disease severity and symptoms across treatments. Table S6. Taxonomic identification of fungal OTUs. Table S7. Fusarium spp. isolation and strain level identification. Fig. S1 Correlation analysis between FocTR4 target gene copies and the Fusarium wilt disease incidence in two independent experiments (indicated as first and second seasons. Correlation analyses were based on Spearman and the P-values were corrected for False Discovery Rate (FDR). Fig. S2 a Principal coordinate analysis (PCoA) of soil bacterial communities based on the Bray-Curtis distances. BS: above-ground banana residue, BR: below-ground banana residue, PS: above-ground pineapple residue, PR: below-ground pineapple residue, and CK: control treatment without residue. ANOSIM: analysis of similarities. b Structural equation model (SEM) linking the microbial (bacterial and fungal) community compositions (based on PCoA), the pathogen FocTR4 density (copy numbers, qPCR), and their respective relationships with the disease incidence. R2, χ2, and P-values denote the fit of the model. c Redundancy analysis (RDA). “***” represents the P < 0.001. AP: rapid available phosphorus, AK: soil available kalium, NO3-: nitrate nitrogen, NH4+: ammonium nitrogen, pH: pH value, EC: electrical conductivity, TOC: total organic carbon, DI: disease incidence, FocRT4: Fusarium oxysporum f. sp. cubense tropical race 4. Fig. S3 Linear discriminant analysis (LDA). a Analysis including below-ground crop residues and the control treatment, and b analysis including above-ground crop residues and the control treatment. BS: above-ground banana residue, BR: below-ground banana residue, PS: above-ground pin [file 40168_2021_1133_MOESM2_ESM.docx]

**Development of fungal-mediated soil suppressiveness against *Fusarium* wilt disease via plant residue manipulation**

Xianfu Yuan^1,2^, Shan Hong^3^, Wu Xiong^1,2^, Waseem Raza^1,2^, Beibei Wang^3^, Rong Li^1,2,^*, Yunze Ruan^3^, Qirong Shen^1,2^, Francisco Dini-Andreote^5,6^

**Authors’ affiliation**

^1^ Jiangsu Provincial Key Lab of Solid Organic Waste Utilization, Jiangsu Collaborative Innovation Center of Solid Organic Wastes, Educational Ministry Engineering Center of Resource-saving fertilizers, Nanjing Agricultural University, Nanjing 210095, Jiangsu, People’s Republic of China

^2^ The Key Laboratory of Plant Immunity, Nanjing Agricultural University, Nanjing 210095, Jiangsu, People’s Republic of China

^3^ Hainan Key Laboratory for Sustainable Utilization of Tropical Bio-resources, College of Tropical Crops, Hainan University, Haikou, 570228, People’s Republic of China

^4^ Ecology and Biodiversity Group, Department of Biology, Institute of Environmental Biology, Utrecht University, 3584 CH Utrecht, the Netherlands

^5^ Department of Plant Science, The Pennsylvania State University, University Park, PA, USA

^6^ Huck Institutes of the Life Sciences, The Pennsylvania State University, University Park, PA, USA

**Supplementary Information**

**Materials and methods for bacterial communities**

One soil DNA sample was selected randomly from each block contained three samples, and a total of three samples were obtained for each treatment. Sequencing libraries were constructed as previously described [1-2], and primers of 515F/806R were used to amplify the V4 region of bacterial 16S rRNA gene using the Thermo Scientific® Phusion High-Fidelity Polymerase Chain Reaction (PCR) Master Mix (New England Biolabs, UK). Details on the amplification protocol are described by Shen et al. [3]. Amplicon libraries were sequenced on an Illumina MiSeq 2000 platform at the Personal Biotechnology Company (Shanghai, China). Bioinformatic analysis was performed as described for fungi in the main text. All raw sequence data are available at the National Center for Biotechnology Information (NCBI) Sequence Read Archive (SRA) database under the accession number PRJNA670608. Structural equation model (SEM) was used to visualize the potential relationships of microbial (fungal and bacteria) composition (based on PCoA), abundance of *Foc*TR4, and banana disease incidence using the R package *sem* [4].

**Supplementary Tables**

**Table S1.** Nutrient content (g/kg) present in the pineapple and banana residues

| Residue | Pineapple | | | | | Banana | | | |
| --- | --- | --- | --- | --- | --- | --- | --- | --- | --- |
|  | N | P_2_O_5_ | K_2_O | MgO | N | | P_2_O_5_ | K_2_O | MgO |
| Above-ground residues | 9.02 | 1.97 | 23.99 | 0.26 | 17.56 | | 4.37 | 72.21 | 1.57 |
| Below-ground residues | 4.28 | 0.98 | 16.26 | 0.18 | 5.39 | | 1.17 | 22.41 | 0.24 |

**Table S2.** Nutrient content corrections (g/pot) carried out across treatments

| Treatment | CO(NH_2_)_2_ | Ca(H_2_PO_4_)_2_ | K_2_SO_4_ |
| --- | --- | --- | --- |
| Pineapple above-ground residue | 1.83 | 5.12 | 8.74 |
| Pineapple below-ground residue | 0.24 | 0.39 | 1.12 |
| Control (CK) | 3.76 | 9.31 | 13.09 |

**Table S3.** Primers and probe sequences used in this study

| **Target** | **Primer or probe** | **Sequence (5’ – 3’)** |
| --- | --- | --- |
| [***A.***](C:/Program%20Files%20(x86)/Youdao/Dict/8.8.1.0/resultui/html/index.html#/javascript:;) [***fumigatus***](C:/Program%20Files%20(x86)/Youdao/Dict/8.8.1.0/resultui/html/index.html#/javascript:;) | *A. fumigatus*-1 | GCCCGCCGTTTCGAC |
|  | AfumiP1^1*^ | CCCGCCGAAGACCCCAACATG |
|  | *A. fumigatus*-2 | CCGTTGTTGAAAGTTTTAACTGATTAC |
| ***Foc*TR4** | FocSc-1 | CAGGGGATGTATGAGGAGGCTAGGCTA |
|  | FocSc-2 | GTGACAGCGTCGTCTAGTTCCTTGGAG |
| ***F. solani*** | Fs-F | GCTTATCTCGGGTCGTGGAA |
|  | Probe2^2*^ | TCATCAGTCACTTCATGCTGA |
|  | Fs-R | CAAGTGACCGGTCTGTAGATGAT |
| **ITS** | ITS1 | TCCGTAGGTGAACCTGCGG |
|  | ITS4 | TCCTCCGCTTATTGATATGC |
| ***Foc*TR4** | *Foc*TR4-F | CACGTTTAAGGTGCCATGAGAG |
|  | *Foc*TR4-R | CGCACGCCAGGACTGCCTCGTGA |
| ***Foc*TR4 (VCG 01213/16)** | 01213/16 F1 | ACG TTTAAGGTGCCATGAGAG |
|  | 01213/16 R2 | CCT CGTGAGCCACTTTTTAT |
| **TEF-1a** | EF1-F | ATGGGTAAGGARGACAAGAC |
|  | EF2-R | GGARGTACCAGTSATCATGTT |

^1*^ The probe contains a 5’ FAM reporter and 3’ dark quencher.

^2*^ The probe contains a 5’ FAM reporter and 3’ MGB quencher.

**Table S4.** Proportion of culturable *Fusarium* spp. obtained in each treatment

| **Taxa** | **CK** | **BS** | **BR** | **PS** | **PR** |
| --- | --- | --- | --- | --- | --- |
| *Fusarium luffae* | 1.7% | 6.7% | 5% | 0% | 0% |
| *Fusarium fujikuro* | 3.3% | 6.7% | 8.3% | 0% | 0% |
| *Fusarium proliferatum* | 1.7% | 5% | 6.7% | 0% | 0% |
| *Fusarium oxysporum* | 5% | 8.3% | 10% | 3.3% | 3.3% |
| *Fusarium solani* | 3.3% | 0% | 6.7% | 3.3% | 20% |
| *Fusarium falciform* | 0% | 1.7% | 0% | 3.3% | 0% |
| *Fusarium nectrioides* | 1.7% | 0% | 0% | 1.7% | 1.7% |

**Table S5.** *Fusarium* wilt disease severity and symptoms across treatments

| **Treatment** | **Leaf yellowing** | **Pseudostem splitting** | **Dark brown discoloration of vascular tissues** | **Plant death** |
| --- | --- | --- | --- | --- |
| CK | **++** | **++** | **++** | **++** |
| BS | **+++** | **+++** | **+++** | **++** |
| BR | **+++** | **+++** | **+++** | **++** |
| PS | **++** | **++** | **++** | **+** |
| PR | **++** | **++** | **++** | **+** |

Note: “+” light disease symptoms; “++” moderate disease symptoms; and “+++” severe disease symptoms.

**Table S6.** Taxonomic identification of fungal OTUs

| **OTU** | **Phyla** | **class** | **Order** | **Family** | **Genus** | **Species** |
| --- | --- | --- | --- | --- | --- | --- |
| OTU3 | Ascomycota | Sordariomycetes | Hypocreales | Nectriaceae | *Fusarium* | *Fusarium solani* |
| OTU11 | Ascomycota | Sordariomycetes | Sordariales | Chaetomiaceae | *Humicola* | *unclassified* |
| OTU15 | Ascomycota | Eurotiomycetes | Eurotiales | Aspergillaceae | *Aspergillus* | *Aspergillus fumigatus* |
| OTU22 | Ascomycota | Unclassified | Unclassified | Unclassified | Unclassified | Unclassified |
| OTU7 | Ascomycota | Sordariomycetes | Hypocreales | Nectriaceae | *Fusarium* | *Fusarium delphinoides* |
| OTU8 | Ascomycota | Eurotiomycetes | Eurotiales | Trichocomaceae | *Talaromyces* | unclassified |
| OTU10 | Ascomycota | Eurotiomycetes | Eurotiales | Aspergillaceae | *Penicillium* | unclassified |
| OTU12 | Ascomycota | Sordariomycetes | Sordariales | Chaetomiaceae | *Ovatospora* | *Ovatospora mollicella* |
| OTU19 | unclassified | unclassified | unclassified | unclassified | unclassified | unclassified |
| OTU41 | Ascomycota | Eurotiomycetes | Eurotiales | Aspergillaceae | *Aspergillus* | unclassified |
| OTU47 | Basidiomycota | unclassified | unclassified | unclassified | unclassified | unclassified |
| OTU70 | Ascomycota | Eurotiomycetes | Eurotiales | Aspergillaceae | *Aspergillus* | unclassified |
| OTU73 | Ascomycota | Unclassified | Unclassified | Unclassified | Unclassified | Unclassified |
| OTU199 | Ascomycota | Lecanoromycetes | unclassified | unclassified | unclassified | unclassified |
| OTU258 | Ascomycota | Unclassified | Unclassified | Unclassified | Unclassified | Unclassified |
| OTU30 | unclassified | unclassified | unclassified | unclassified | unclassified | unclassified |
| OTU40 | Ascomycota | Sordariomycetes | Sordariales | Lasiosphaeriaceae | *Cladorrhinum* | *Cladorrhinum bulbillosum* |
| OTU45 | Ascomycota | Lecanoromycetes | unclassified | unclassified | unclassified | unclassified |
| OTU57 | Ascomycota | Pezizomycetes | Pezizales | Ascodesmidaceae | *Cephaliophora* | unclassified |
| OTU76 | Ascomycota | Sordariomycetes | Sordariales | Chaetomiaceae | *Mycothermus* | *Mycothermus thermophilus* |

**Table S7.** *Fusarium* spp. isolation and strain level identification

| **Designation** | **Origin** | **Classification** | **Mycelial**  **feature** | **Macroconidium**  **shape** | **Colony color** |
| --- | --- | --- | --- | --- | --- |
| F1 | Jianfeng | *F. solani* | Arachnoid | Sickle-shaped | White |
| F2 | Jianfeng | *F. fujikuroi* | Flocculence | Sickle-shaped | White |
| F3 | Jianfeng | *F. proliferatum* | Villous | Sickle-shaped | White |
| F4 | Jianfeng | *F. solani* | Arachnoid | Sickle-shaped | White |
| F5 | Jianfeng | *F. oxysporum* | Patcky | Sickle-shaped | Red violet |
| F6 | Jianfeng | *F. oxysporum* | Patcky | Sickle-shaped | Red violet |
| F7 | Jianfeng | *F. oxysporum* | Patcky | Sickle-shaped | Red violet |
| F8 | Jianfeng | *F. oxysporum* | Patcky | Sickle-shaped | Red violet |
| F9 | Jianfeng | *F. oxysporum* | Patcky | Sickle-shaped | Red violet |
| F10 | Jianfeng | *F. oxysporum* | Patcky | Sickle-shaped | Red violet |
| F11 | Jianfeng | *F. oxysporum* | Patcky | Sickle-shaped | Red violet |
| F12 | Jianfeng | *F. oxysporum* | Patcky | Sickle-shaped | Red violet |
| F13 | Jianfeng | *F. oxysporum* | Patcky | Sickle-shaped | Red violet |
| F14 | Jianfeng | *F. oxysporum* | Patcky | Sickle-shaped | Red violet |
| F15 | Jianfeng | *F. oxysporum* | Patcky | Sickle-shaped | Red violet |
| F16 | Jianfeng | *F. oxysporum* | Patcky | Sickle-shaped | Red violet |
| F17 | Jianfeng | *F. oxysporum* | Patcky | Sickle-shaped | Red violet |
| F18 | Jianfeng | *F. oxysporum* | Patcky | Sickle-shaped | Red violet |
| F19 | Jianfeng | *F. oxysporum* | Patcky | Sickle-shaped | Red violet |
| F20 | Jianfeng | *F. oxysporum* | Patcky | Sickle-shaped | Red violet |
| F21 | Jianfeng | *F. oxysporum* | Patcky | Sickle-shaped | Red violet |
| F22 | Jianfeng | *F. oxysporum* | Patcky | Sickle-shaped | Red violet |
| F23 | Jianfeng | *F. oxysporum* | Patcky | Sickle-shaped | Red violet |
| F24 | Jianfeng | *F. oxysporum* | Patcky | Sickle-shaped | Red violet |
| F25 | Jianfeng | *F. oxysporum* | Patcky | Sickle-shaped | Red violet |
| F26 | Jianfeng | *F. oxysporum* | Patcky | Sickle-shaped | Red violet |
| *Foc*4 | Jianfeng | *F. oxysporum* | Patcky | Sickle-shaped | Red violet |

**Supplementary Figures**


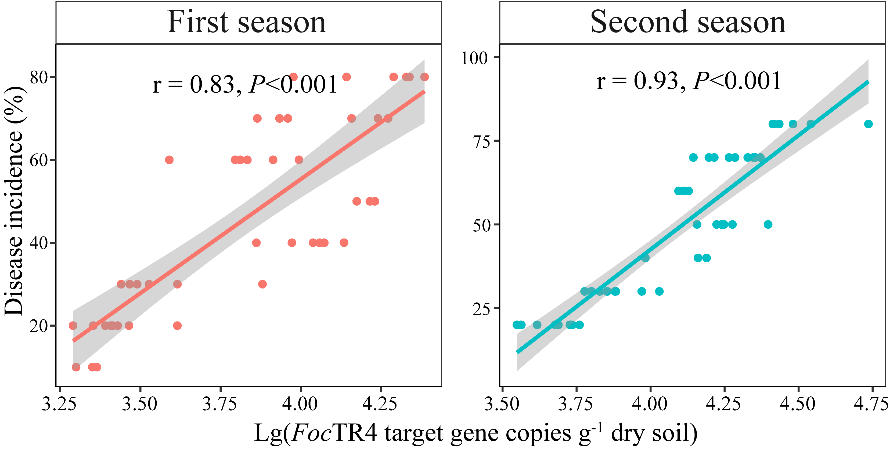


**Fig. S1** Correlation analysis between *Foc*TR4 target gene copies and the *Fusarium* wilt disease incidence in two independent experiments (indicated as first and second seasons. Correlation analyses were based on Spearman and the *P*-values were corrected for False Discovery Rate (FDR).


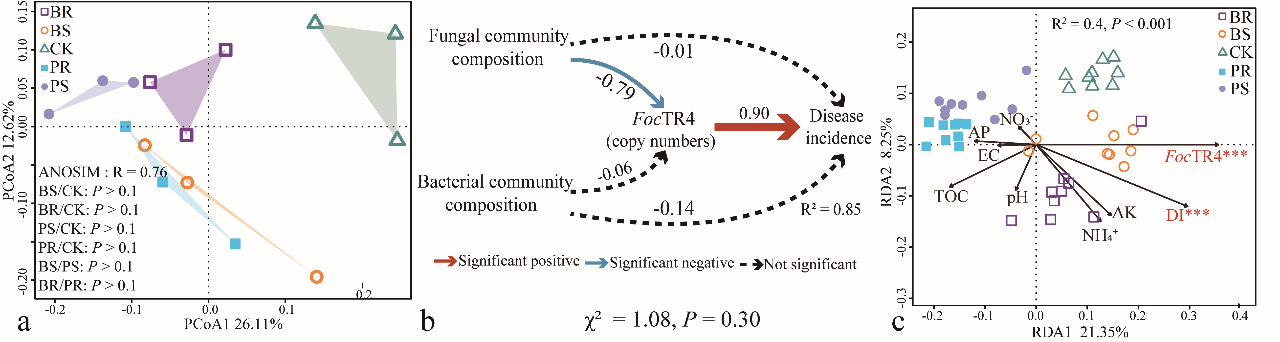


**Fig. S2** **a** Principal coordinate analysis (PCoA) of soil bacterial communities based on the Bray-Curtis distances. BS: above-ground banana residue, BR: below-ground banana residue, PS: above-ground pineapple residue, PR: below-ground pineapple residue, and CK: control treatment without residue. ANOSIM: analysis of similarities. **b** Structural equation model (SEM) linking the microbial (bacterial and fungal) community compositions (based on PCoA), the pathogen *Foc*TR4 density (copy numbers, qPCR), and their respective relationships with the disease incidence. R^2^, χ2, and P-values denote the fit of the model. **c** Redundancy analysis (RDA). “***” represents the *P* < 0.001. AP: rapid available phosphorus, AK: soil available kalium, NO_3_^-^: nitrate nitrogen, NH_4_^+^: ammonium nitrogen, pH: pH value, EC: electrical conductivity, TOC: total organic carbon, DI: disease incidence, *Foc*RT4: *Fusarium oxysporum* f. sp. *cubense* tropical race 4.


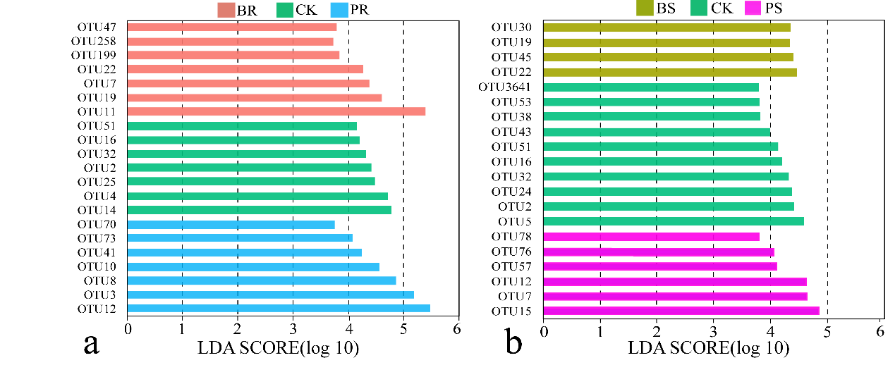


**Fig. S3** Linear discriminant analysis (LDA). **a** Analysis including below-ground crop residues and the control treatment, and **b** analysis including above-ground crop residues and the control treatment. BS: above-ground banana residue, BR: below-ground banana residue, PS: above-ground pineapple residue, PR: below-ground pineapple residue, and CK: control treatment without residue.


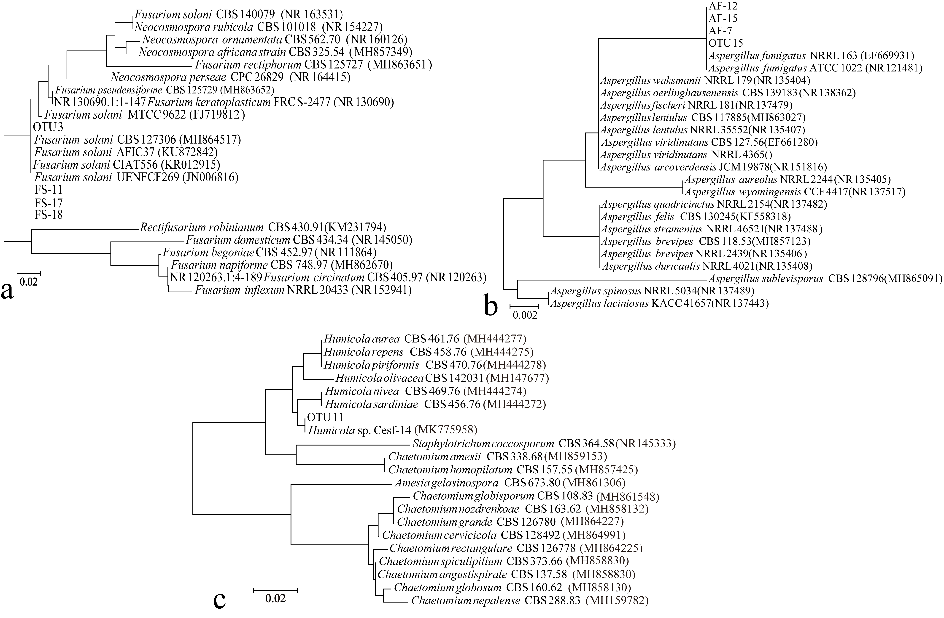


**Fig. S4** Phylogenetic reconstructions of **a** *F. solani* isolates (FS11, FS17, and FS18) and OTU3, **b** *A. fumigatus* isolates (AS-7, AS-12, and AS-15) and OTU15, and **c** OTU11. Each independent phylogenetic reconstruction included best match sequences obtained from the NCBI database for taxonomical inferences.


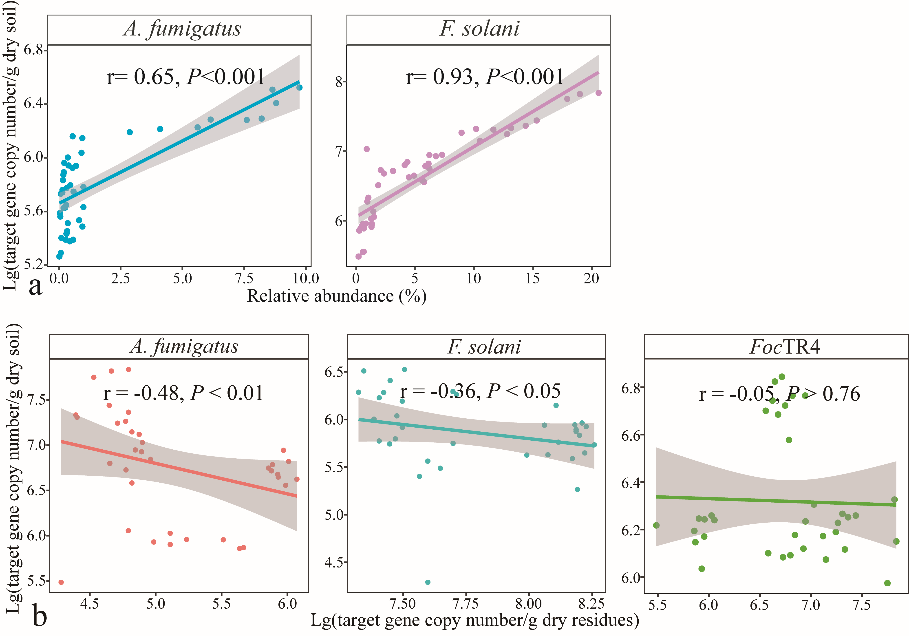


**Fig. S5** **a** Correlation plots displaying the relationships between the target gene copy numbers (qPCR) of specific fungal taxa (*A. fumigatus* and *F. solani*) in soil and their respective relative abundances in soil obtained by Illumina Miseq sequencing. **b** Correlation plots displaying the relationships between the target gene copy numbers (qPCR) of specific fungal taxa in crop residues (*A. fumigatus* and *F. solani*) and the target gene copy numbers (qPCR) of specific fungal taxa in soil. Correlation analyses were based on Spearman and the *P*-values were corrected for False Discovery Rate (FDR).


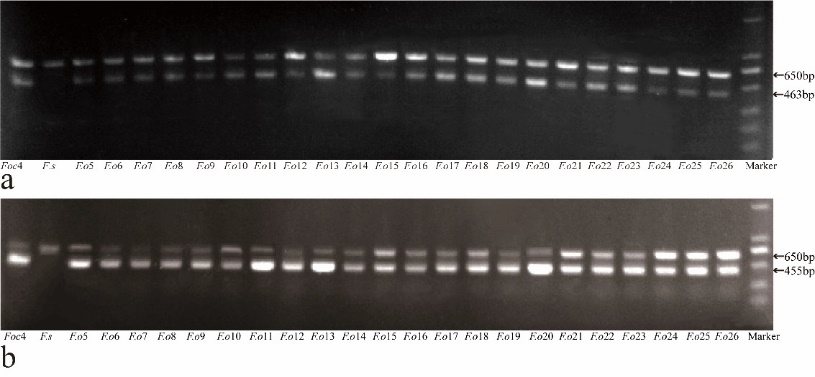


**Fig. S6 a** Results from the multiplex-PCR system based on the targets TEF-1α (650bp, positive control) and *Foc*TR4 (463bp). **b** Results from the multiplex-PCR system based on the targets TEF-1α (650bp, positive control) and *Foc*TR4 (VCG 01213/16) (455bp). *F.s*: *F. solani* (negative control).

**
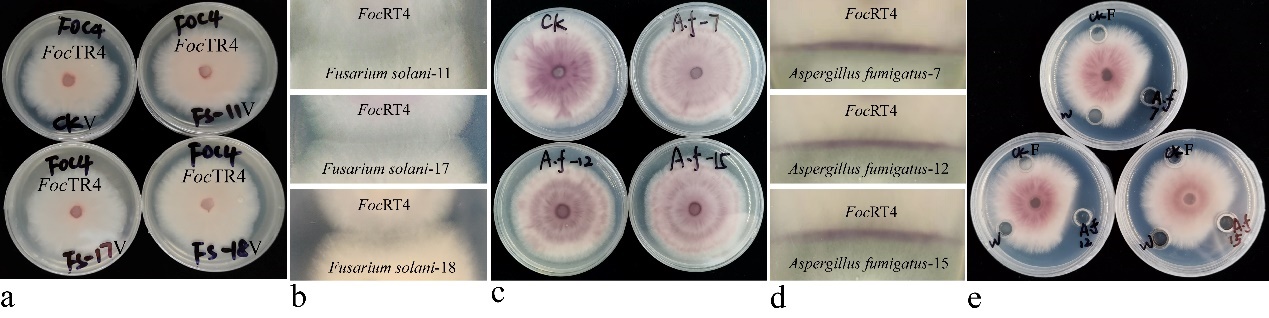
**

**Fig. S7** Results obtained from the co-culture experiments to test the potential of *F. solani* and *A. fumigatus* isolates in inhibiting the colony growth of the pathogen *Foc*TR4. **a, b** Display the potential effects on *Foc*TR4 colony growth mediated by volatile compounds and secreted substances produced by *F. solani*. **c, d** Display the potential inhibiting effects on *Foc*TR4 colony growth mediated by volatile compounds and secreted substances produced by *A. fumigatus*. **e** Antagonistic effects of sterile fermentation fluid from *A. fumigatus* on *Foc*RT4, CK: added water, W: added fluid medium, A.F (7, 12,15): added sterile fermentation fluid from *A. fumigatus*.


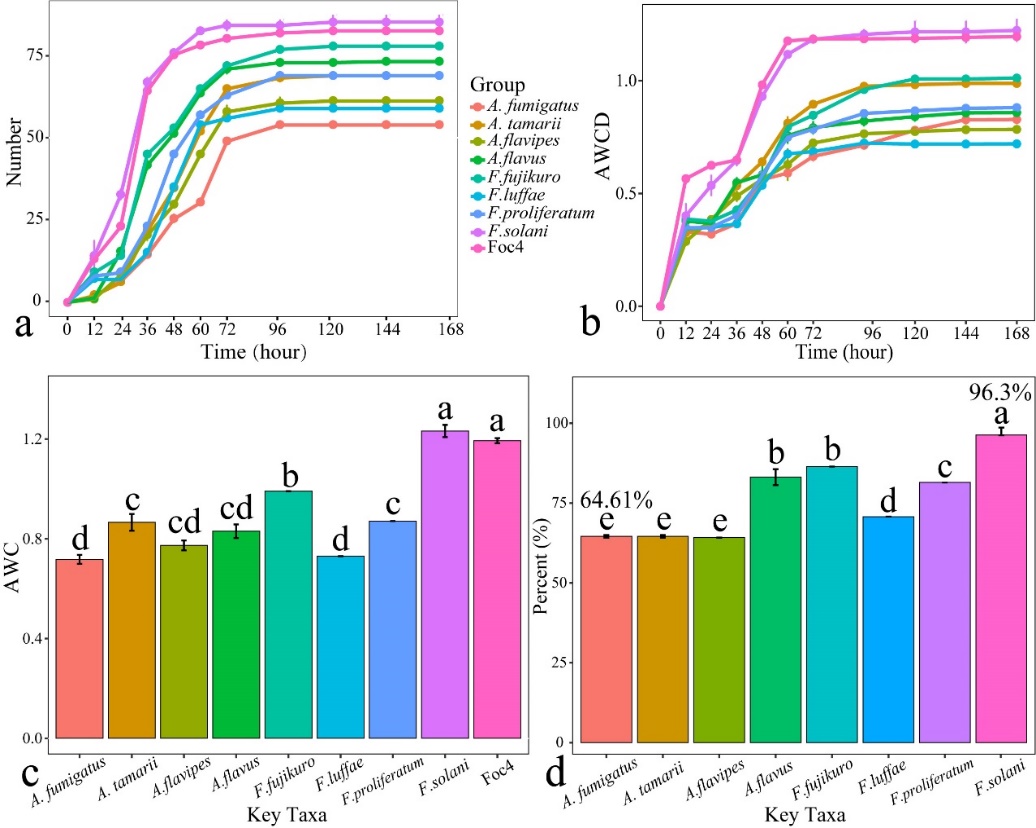


**Fig. S8** **a** Number of carbon source utilization by each individual fungal taxa thought time. **b** Carbon source metabolic rate of each fungal taxa indicated by the average well color development (AWCD). **c** Average well color (AWC) values of common carbon source utilization between *Foc*TR4 and each individual fungal taxa tested at the 96 hours time point. **d** Percentage of common carbon source utilization between *Foc*TR4 and each individual fungal taxa tested at the 96 hours time point. Different lowercase letters indicate statistically significant differences (*P* < 0.05) according to Tukey's HSD test.


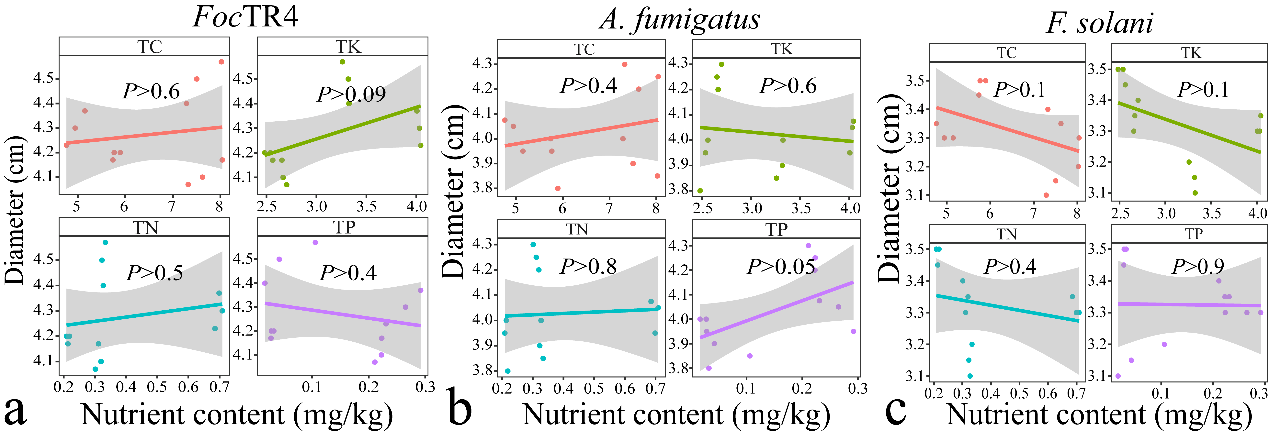


**Fig. S9** Correlation analyses between the colony growth (in diameter) of each specific fungal taxa and the nutrient contents (mg/kg) in the residue extracts. **a** Pathogen *Foc*TR4, **b** *A. fumigatus*, and **c** *F. solani*. TN: total nitrogen, TC: total carbon, TP: total phosphorus, TK: total kalium. Correlation analyses were based on Spearman and the *P*-values were corrected for False Discovery Rate (FDR).


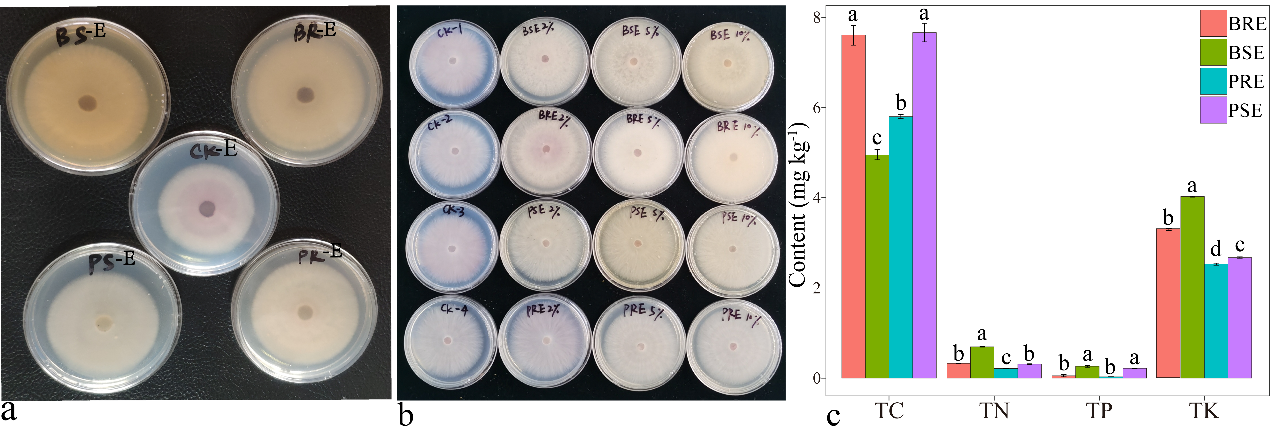


**Fig. S10** Visualization of the colony growth of the pathogen *Foc*TR4 on media supplemented with 2% **(a)** and 2%, 5% and 10% **(b)** of plant reside extracts (BSE, BRE, PSE, PRE). **c** Nutrient contents (mg/kg) in each crop reside extract. BSE: above-ground banana residue extract, BRE: below-ground banana residue extract, PSE: above-ground pineapple residue extract, PRE: below-ground pineapple residue extract, CKE: control using sterile deionized water. TN: total nitrogen, TC: total carbon, TP: total phosphorus, TK: total kalium. Different lowercase letters indicate statistically significant differences (*P* < 0.05) according to Tukey's HSD test.


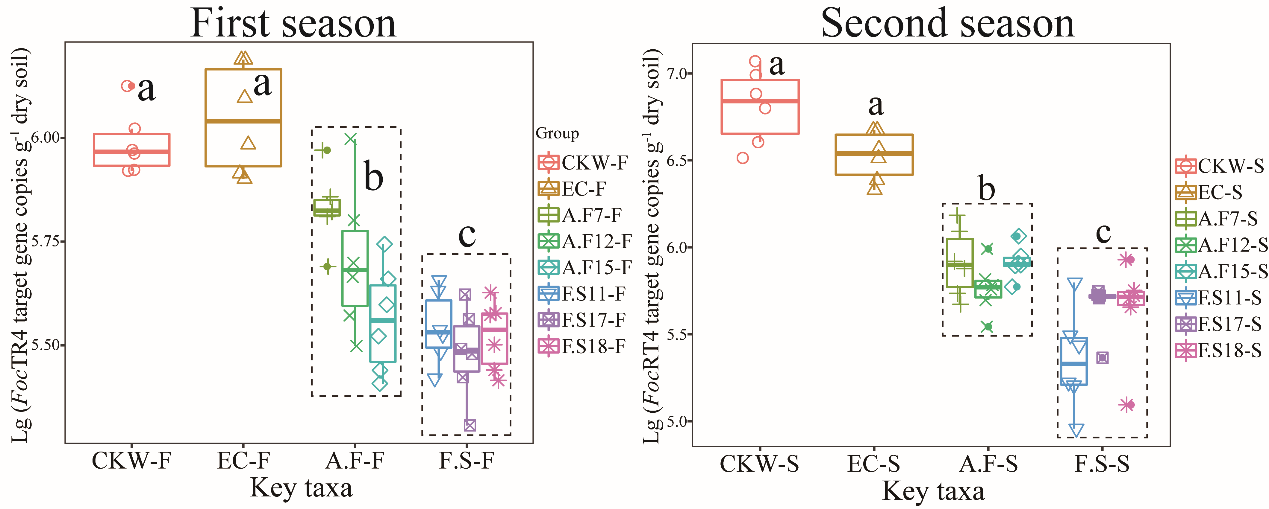


**Fig. S11** Absolute abundance of *Foc*TR4 target gene copies in soil across different inoculation treatments. **a** first season and **b** second season (two independent experiments). CKW-F: control inoculated with sterile water, EC-F: control inoculated with *Escherichia coli*, A.F-F: inoculation with a combination of three *A. fumigatus* isolates (A.F-7, A.F-12, and A.F-15), F.S-F: inoculation with a combination of three *F. solani* isolates (F.S-11, F.S-17, and F.S-18). Different lowercase letters indicate statistically significant differences (*P* < 0.05) according to Tukey's HSD test.

**References**

1. Caporaso JG, Lauber CL, Walters WA, Berg L-D, Lozupone CA, Turnbaugh PJ, Fierer N, Knight R. Global patterns of 16S rRNA diversity at a depth of millions of sequences per sample. P Natl Acad Sci USA. 2011; 108: 4516-4522.

2. Kozich JJ, Westcott SL, Baxter NT, Highlander SK, Schloss PD. Development of a dual-index sequencing strategy and curation pipeline for analyzing amplicon sequence data on the MiSeq Illumina sequencing platform. Appl Environ Microb. 2013; 79(17): 5112-5120.

3. Shen Z, Xu e C, Penton CR, Thomashow LS, Zhang N, Wang B, Ruan Y, Li R, Shen Q. Suppression of banana Fusarium wilt disease induced by soil microbiome reconstruction through an integrated agricultural strategy. Soil Biol Biochem. 2019; 128: 164-174.

4. Mamet SD, Redlick E, Brabant M, Lamb EG, Helgason BL, Stanley K, Siciliano SD. Structural equation modeling of a winnowed soil microbiome identifies how invasive plants re-structure microbial networks. ISME J. 2019; 13:1988-1996.
